# Platelet-Derived Short-Chain Polyphosphates Enhance the Inactivation of Tissue Factor Pathway Inhibitor by Activated Coagulation Factor XI

**DOI:** 10.1371/journal.pone.0165172

**Published:** 2016-10-20

**Authors:** Cristina Puy, Erik I. Tucker, Ivan S. Ivanov, David Gailani, Stephanie A. Smith, James H. Morrissey, András Gruber, Owen J. T. McCarty

**Affiliations:** 1 Departments of Biomedical Engineering Oregon Health & Science University, Portland, Oregon, United States of America; 2 Division of Hematology and Medical Oncology, School of Medicine, Oregon Health & Science University, Portland, Oregon, United States of America; 3 Aronora, Inc, Portland, Oregon, United States of America; 4 Departments of Pathology and Medicine, Vanderbilt University School of Medicine, Nashville, Tennessee, United States of America; 5 Biochemistry Department, University of Illinois, Urbana, Illinois, United States of America; 6 Department of Cell, Developmental and Cancer Biology Oregon Health & Science University, Portland, Oregon, United States of America; Ludwig-Maximilians-Universitat Munchen, GERMANY

## Abstract

**Introduction:**

Factor (F) XI supports both normal human hemostasis and pathological thrombosis. Activated FXI (FXIa) promotes thrombin generation by enzymatic activation of FXI, FIX, FX, and FV, and inactivation of alpha tissue factor pathway inhibitor (TFPIα), *in vitro*. Some of these reactions are now known to be enhanced by short-chain polyphosphates (SCP) derived from activated platelets. These SCPs act as a cofactor for the activation of FXI and FV by thrombin and FXIa, respectively. Since SCPs have been shown to inhibit the anticoagulant function of TFPIα, we herein investigated whether SCPs could serve as cofactors for the proteolytic inactivation of TFPIα by FXIa, further promoting the efficiency of the extrinsic pathway of coagulation to generate thrombin.

**Methods and Results:**

Purified soluble SCP was prepared by size-fractionation of sodium polyphosphate. TFPIα proteolysis was analyzed by western blot. TFPIα activity was measured as inhibition of FX activation and activity in coagulation and chromogenic assays. SCPs significantly accelerated the rate of inactivation of TFPIα by FXIa in both purified systems and in recalcified plasma. Moreover, platelet-derived SCP accelerated the rate of inactivation of platelet-derived TFPIα by FXIa. TFPIα activity was not affected by SCP in recalcified FXI-depleted plasma.

**Conclusions:**

Our data suggest that SCP is a cofactor for TFPIα inactivation by FXIa, thus, expanding the range of hemostatic FXIa substrates that may be affected by the cofactor functions of platelet-derived SCP.

## Introduction

Factor (F) XI was originally described in the waterfall model of coagulation as part of the intrinsic or contact pathway, but it appears to be the only contact factor that contributes to hemostasis. Congenital deficiency of FXI is occasionally associated with trauma-associated abnormal bleeding, especially in tissues with increased fibrinolytic activity [1]. Since FXII, prekallikrein and high molecular weight kininogen deficiencies do not affect hemostasis, the hemostatic function of FXI may well be manifested through feedback activation by thrombin generated by exposure of blood to tissue factor (TF) [2,3]. FXIa can also promote thrombin generation through direct activation of FX, FV and FVIII [4–6]. Additionally, activated platelets release polyphosphates, with chain lengths of ~70–100 phosphate units, which are polyanionic macromolecules that accumulate in cells, and can support catalytic reactions. Similar to some other polyanions, short-chain polyphosphates (SCP) enhance the feedback activation of FXI by thrombin approximately three thousand-fold [7,8]. Also, platelet-derived SCP have been shown to enhance FV activation by FXIa [9], suggesting that in the presence of activated platelets, FXIa may support hemostasis even in the absence of FIX.

TF pathway inhibitor alpha (TFPIα), the anticoagulant isoform of the alternatively spliced plasma protease inhibitor TFPI [10], reversibly inhibits FXa through the Kunitz 2 (K2) domain of TFPI, and, in a FXa-dependent manner, the TF-FVIIa complex through the Kunitz 1 (K1) domain [11,12], whereas the Kunitz 3 (K3) domain has no known inhibitory function. TFPI is required for proper embryonic development and hemostasis. Reduced plasma TFPI levels have been shown to reverse the hemorrhagic defect and prolong the survival of TF-null transgenic mice expressing a low level of human TF [13]. We recently demonstrated that FXIa binds to TFPIα, and inhibits its anticoagulant activity by cleaving TFPIα between the Kunitz-type domains K1 and K2 (Lys86/Thr87) and also at the active sites of the K2 (Arg107/Gly108) and K3 (Arg199/Ala200) domains, thereby accelerating thrombin generation [14].

Polyphosphates, including SCP, have been shown to antagonize the anticoagulant activity of TFPIα on TF-induced clotting time in plasma via an incompletely defined mechanism [15]. Polyphosphates of various lengths, including SCP of the size-range secreted by activated human platelets, are able to block the anticoagulant activity of TFPIα [16]. Polyanions, including polyphosphates, have been shown to enhance several enzymatic reactions involving FXI or FXIa [17]. Along these lines, the A3 and catalytic domains of FXI contain anion-binding sites (ABS), which are required for heparin-mediated enhancement of FXIa inhibition by antithrombin and for SCP-mediated enhancement of FXI activation [18]. For human FXI, Lys529, Arg530, and Arg532 in the catalytic domain form one of the ABS. Here we show that SCP binds TFPIα and acts as a cofactor for the cleavage and inactivation of TFPIα by FXIa in both a purified system and in plasma, identifying yet another molecular mechanism by which platelets and FXI may reciprocally and in concert support hemostatic thrombin generation.

## Materials and Methods

### Reagents

Soluble SCP (~70–100 phosphate units in length) were prepared by size-fractionation of sodium polyphosphate by preparative polyacrylamide gel electrophoresis as previously described (16). A heterogeneous polyphosphate preparation, comprising 50 to 1000 phosphate units in length, was biotinylated on terminal phosphates (bio-polyP) using amine-PEG2-biotin from Pierce Chemical as described previously (7). Polyphosphate binding protein (PPxbd) was prepared as previously described [19]. The anti-FXI monoclonal antibody, 1A6, that blocks the activation of FIX and FV by FXIa [6] but not the activation of FX [4] or inactivation of TFPIα by FXIa [14], was generated as previously described [2]. The anti-FXI monoclonal antibody, 10C9, which binds near the FXIa active site and inhibits FXIa cleavage of a chromogenic substrate, was generated as previously described [14]. Neutralizing monoclonal mouse anti-human TFPI antibodies, specific for the K1 or K2 domains, were from MyBiosource (San Diego, CA, USA). Recombinant full-length TFPIα was a kind gift from Dr. George J. Broze (Washington University, St. Louis, MO, USA). Chromogenic substrate for FXa (Spectrozyme® FXa) was from American Diagnostica, Inc. (Stamford, CT, USA; now Sekisui Diagnostics). Dade® Innovin® Reagent was used as a source of recombinant TF (Siemens Healthcare Diagnostics, Deerfield, IL, USA). The thrombin inhibitor, hirudin, the calcium ionophore, A23187, *o*-phenylenediamine dihydrochloride (OPD) substrate and the protease inhibitor, aprotinin, were from Sigma-Aldrich (St Louis, MO, USA). Collagen-related peptide (CRP) was from R. Farndale (Cambridge University, UK). Plasma-derived FVIIa, FX, FXa, FX-depleted plasma, FXI-depleted plasma and FIX-depleted plasma were from Haematologic Technologies, Inc (Essex Junction, VT, USA). Streptavidin-horseradish peroxidase (HRP) conjugate was from Thermo Fisher Scientific (Grand Island, NY, USA). Recombinant human FXI was expressed in HEK293 fibroblasts and purified from conditioned media and characterized as described [18]. Wild-type FXI (FXI^WT^) and a FXI species with alanine substitutions for Lys529, Arg530 and Arg532 (FXI^ABS^) were prepared as described [18]. Lys529, Arg530 and Arg532 form an anion-binding site (ABS) that mediates the interaction of FXI with polyanions, including heparin, dextran sulfate, or polyphosphate. The alanine substitutions were introduced to determine if the ABS has an effect on the proteolysis of TFPIα in the presence or absence of SCP. FXI (2μM) was converted to FXIa by incubation with 0.05 μM FXIIa in 50 mM Tris-HCl pH 7.4, 100 mM NaCl (TBS). Activated proteins were frozen and stored at -80°C.

### Ethics Statement

All human blood donors provided written informed consent in accordance with a protocol approved by the Oregon Health & Science University Institutional Review Board.

### Western blotting

For TFPI detection by Western blot, rTFPI or supernatant from activated platelets were subject to SDS-PAGE in non-reducing sample buffer and transferred to PVDF membranes and immunoblotted with an anti-TFPI antibody and HRP-conjugated secondary antibodies. Proteins were detected using ECL (GE Healthcare, Piscataway, NJ, USA). Densitometric analysis of images of protein bands was done using ImageJ software.

### Measurement of polyphosphate binding to TFPIα

200 μl of TFPIα (5 μg/ml) was added to microplate wells (Sigma-Aldrich) and incubated for 1hr, followed by blocking with 1% BSA for 2 hrs prior to addition of bio-polyP in 100 μl 25mM HEPES, pH 7.4, 150 mM NaCl (HBS), 0.05% polysorbate 20 (Tween 20) and 1% bovine serum albumin (BSA). After 30 min, the plate was washed three times with HBS and 0.05% Tween-20. Binding was detected with a streptavidin-HRP (1:2000) in a blocking buffer and measured with an OPD substrate at 492 nm. In selected experiments, non-biotinylated SCP (100 μM) was added before the addition of bio-polyP.

### Amidolytic assay to measure FXa generation in purified systems

Dade® Innovin® Reagent stock solution containing lyophilized lipidated TF (5 nM) [20] for prothrombin time testing was dissolved as instructed by the manufacturer, then diluted to a TF concentration of 50 pM with HBS that contained 5 mM CaCl_2_, (HBS-Ca^2+^) and 0.3% BSA. TF was then incubated with 50 pM FVIIa and 100 nM FX in the presence of increasing concentrations of TFPIα (final volume 100 μl) for 10 min. 20 μL HBS containing 100 mM EDTA was added to stop the reaction. Spectrozyme Xa substrate (0.3 mM) was added and the rate of its hydrolysis was measured at 405 nm and converted to FXa concentrations using a standard curve. In some experiments, TFPIα (10 nM) was incubated with FXIa (2 nM) in the absence or presence of SCP (10 μM), with or without Zn^2+^ (25 μM) for 30 min. Samples were quenched with polybrene (16 μM) to neutralize polyphosphates, and aprotinin (50 μM) to inactivate FXIa.

### Preparation of supernatant from activated platelets

Human venous blood was drawn in accordance with an IRB-approved protocol from healthy donors and platelets were purified as previously described [21]. Platelets at a concentration of 2×10^8^ platelets/ml were stimulated with 1 nM thrombin and the Ca^2+^ ionophore A23187 (2 μM) in HBS-Ca^2+^ and 0.3% BSA for 15 min at 37°C to induce degranulation and release of platelet-derived short-chain polyphosphate (SCP) [7] and TFPIα [22]. Subsequently, 1 μM hirudin was added to neutralize thrombin. Platelets were then removed from the suspension by centrifugation at 1000g for 10 min, the supernatant was removed and subjected to 15,000g for 10 min, and 20 μl of the supernatant was used as a source for SCP and TFPIα in an assay to measure the inhibition of FXa generation by TF-FVIIa. Selected experiment were done in the presence of supernatant from platelets activated with either thrombin or thrombin and the platelet GPVI receptor-specific agonist, collagen-related peptide (CRP) (10 μg/ml). Supernatant from resting platelets, prepared in a similar manner but without addition of thrombin or calcium ionophore served as negative control in the FXa generation assay.

### TF- and recalcification-initiated coagulation assay in plasma

Human platelet-poor plasma (PPP) was prepared and clotting time measured as previously described [4]. Plasma (50 μl) was incubated with HBS, or 5 μM SCP (50 μl) in HBS, for 3 min at 37°C in coagulometer cuvettes. Subsequently, 50 μl of CaCl_2_ (8.3 mM final) and diluted Innovin (8 pM final TF concentration) were added, and time to clot formation was measured with a KC4 Coagulation Analyzer (Trinity Biotech, Ireland). In selected experiments, FXI- or FIX-depleted PPP was used.

### FXa- and recalcification-initiated coagulation assay in plasma

In order to determine whether SCP enhances the ability of FXIa to abrogate the anticoagulant activity of TFPIα in plasma, 0.5 nM FXa was added to FX-depleted plasma in the presence of the anti-FXI antibody, 1A6, which inhibits the activation of FV by FXIa and the activation of FIX by FXIa, but does not inhibit the inactivation of TFPIα by FXIa or the activation of FX by FXIa. FX-depleted plasma (50 μl) was incubated for 3 min in the presence of vehicle or 5 nM TFPIα (50 μl) in HEPES-buffered saline at 37°C in coagulometer cuvettes. Subsequently, 50 μl of CaCl_2_ (8.3 mM final) and FXa (0.5 nM) were added, and time to clot formation was measured. In some experiments, TFPIα (5 nM) was incubated with FXIa (1 nM) in the absence or presence of SCP (10 μM) and/or Zn^2+^ (25 μM). Samples were quenched with polybrene (16 μM) to neutralize polyphosphates, and aprotinin (50 μM) to inactivate FXIa, before adding to plasma.

## Results

### Short-chain polyphosphates in the presence of Zn^2+^accelerate the inhibition of TFPIα by FXIa

**Fig 1:**
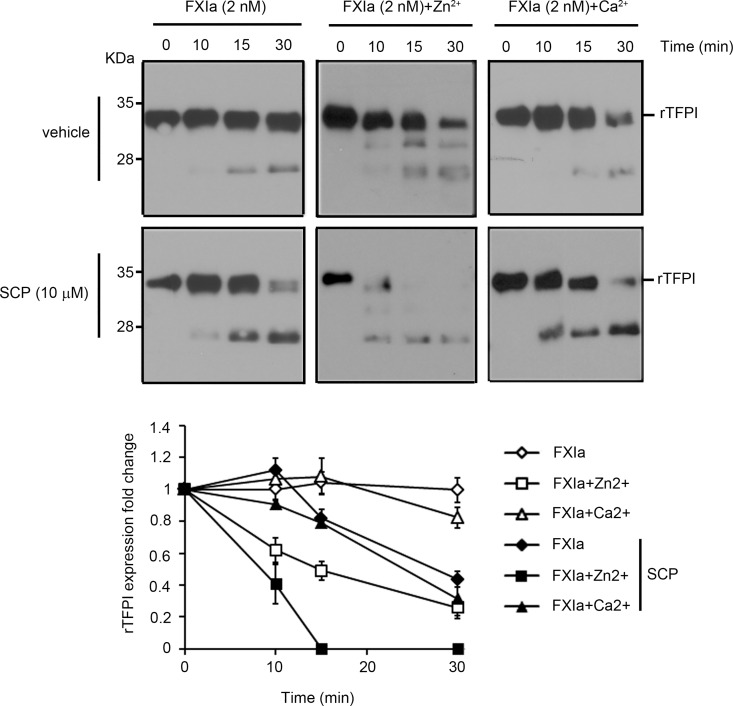
Short-chain polyphosphates accelerate the inhibition of TFPIα by FXIa. rTFPI (10 nM) was incubated with 2 nM FXIa for 10, 15, or 30 min in the presence or absence of 25 μM Zn^2+^,5 mM Ca^2+^, 10 μM SCP, Ca^2+^ and SCP or Zn^2+^ and SCP. rTFPI was analyzed by western blotting, with a polyclonal anti-TFPI antibody under non-reducing conditions. The bands were quantified by densitometry. Data are mean ± SE (n = 3).

Experiments were designed in order to study whether SCPs enhance the rate of TFPI proteolysis by FXIa. Western blot analyses of rTFPI under non-reducing conditions with a polyclonal anti-TFPI antibody demonstrated that the incubation of rTFPI with 2 nM FXIa in the presence of SCP led to the disappearance of the broad upper band and the appearance of a lower molecular mass band, indicating that SCP increases the rate of TFPI proteolysis by FXIa (Fig 1). In a manner similar to our previous findings [14], the presence of Zn^2+^, but not Ca^2+^, increased the rate of TFPI proteolysis by FXIa. Here we found that the presence of SCP together with Zn^2+^ further increased the rate of TFPI proteolysis by FXIa, leading to the complete disappearance of TFPI after 15 min (Fig 1). In contrast, the presence of Ca^2+^ did not increase the rate of TFPI proteolysis by FXIa in the presence of SCP.

### Short-chain polyphosphates bind TFPI

**Fig 2:**
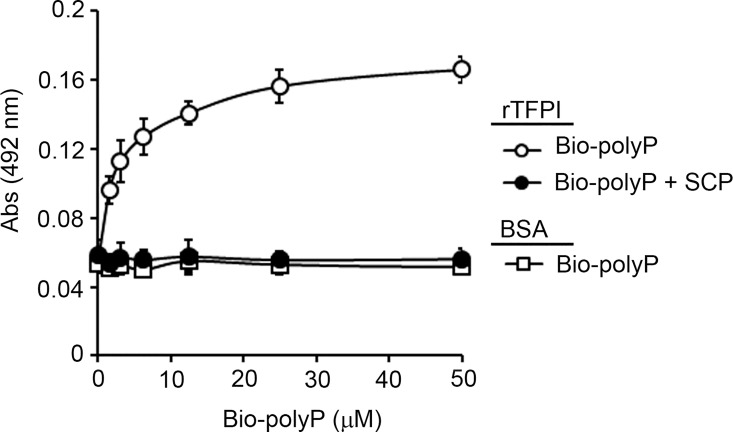
Characterization of the interaction between polyphosphate and TFPIα. (**A**) 96-well plates were coated with 5 μg/ml TFPIα (○,●) or BSA (◻) and increasing concentrations of biotinylated-polyphosphate (bio-polyP) (◻, ○,●) was added to selected wells. Selected experiments were done in the presence of 100 μM SCP (●). Binding was detected with streptavidin-HRP. Data are mean ± SE (n = 3).

To study whether short-chain polyphosphate binds TFPIα, bio-polyP (0–50 μM) was incubated in a microtiter plate over immobilized TFPIα and the level of bound bio-polyP was determined using HRP-streptavidin. As shown in Fig 2, bio-polyP bound to immobilized TFPIα in a concentration dependent-manner with an apparent K_D_ of 1.31 μM, while bio-polyP did not bind to immobilized BSA. The incubation of 100 μM SCP (Fig 2) or heparin (S1 Fig) inhibited the binding of bio-polyP to immobilized TFPIα.

### Short-chain polyphosphates promote TF/FVIIa-induced FX activation in the presence of TFPIα and FXIa

**Fig 3:**
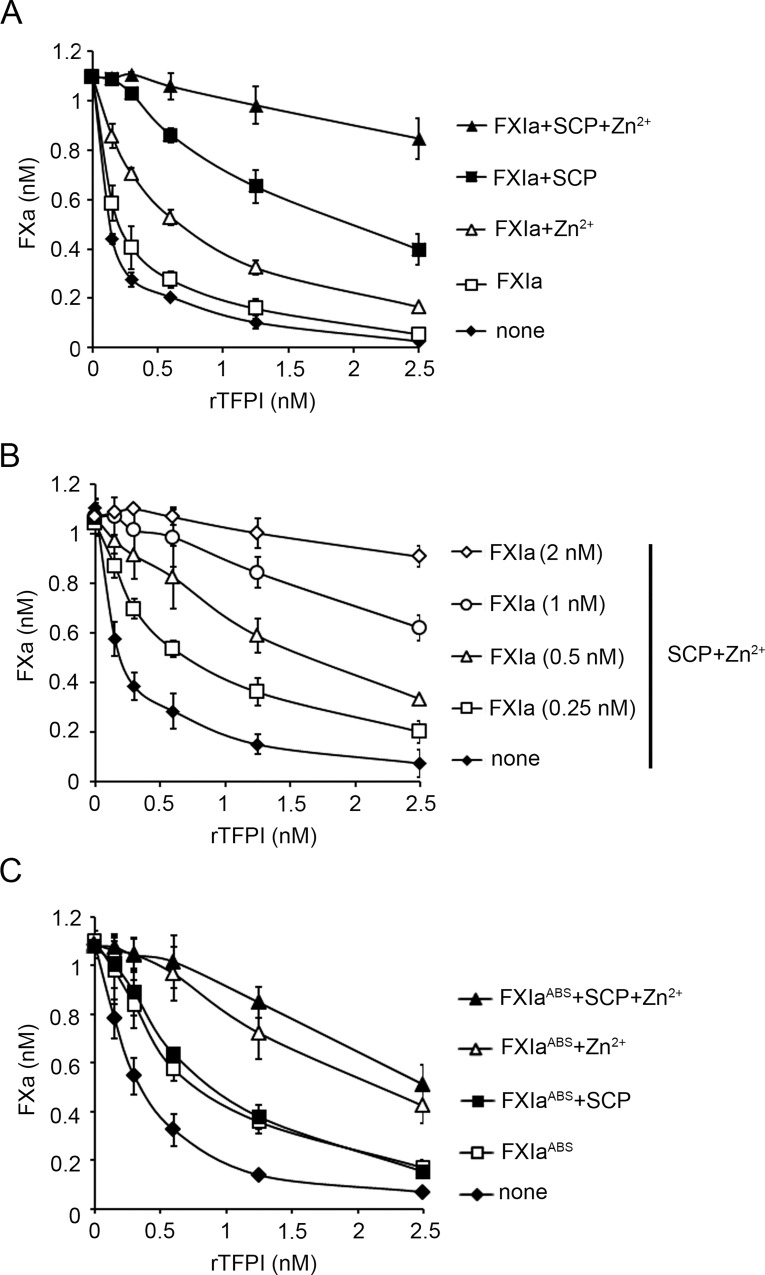
Short-chain polyphosphates accelerate the inhibition of TFPIα by FXIa. (**A**) TFPIα (10 nM) was pretreated with FXIa (2 nM) in the absence or presence of 25 μM Zn^2+^, 10 μM platelet-size polyphosphate (SCP), or Zn^2+^ and SCP. After 30 min of incubation with FXIa, aprotinin (50 μM) and polybrene (16 μM) were added to all samples to stop the reaction. FXa generation by the TF-FVIIa complex in the presence of different concentrations of TFPIα was measured. (**B**) TFPIα (10 nM) was pretreated with different concentrations of FXIa in the presence of 25 μM Zn^2+^ and 10 μM SCP. After 30 min of incubation with FXIa, aprotinin (50 μM) and polybrene (16 μM) were added to all samples to stop the reaction and FXa generation by the TF-FVIIa complex in the presence of different concentrations of TFPIα was measured. (**C**) TFPIα (10 nM) was pretreated with 4 nM FXIa^ABS^ in the presence of 25 μM Zn^2+^, 10 μM SCP, or Zn^2+^ and SCP. After 30 min of incubation with FXIa^ABS^, aprotinin (50 μM) and polybrene (16 μM) were added to all samples to stop the reaction and FXa generation by the TF-FVIIa complex in the presence of different concentrations of TFPIα was measured. Data are mean ± SE (n = 3).

In order to determine whether SCPs act as a cofactor for the proteolysis of TFPIα by FXIa, we measured the effect of TFPIα on the rate of FX activation by the TF-FVIIa complex in a purified system. The addition of increasing concentrations of TFPIα (0–2.5 nM) reduced the generation of FXa from the baseline of 1.09±0.01 nM to 0.03±0.009 nM in a concentration-dependent manner (Fig 3A). After preincubation of TFPIα with 2 nM FXIa for 30 min, the addition TFPIα reduced the generation of FXa from the baseline of 1.09±0.01 nM to 0.05±0.009 pmoles/min, slightly inhibiting the function of TFPIα. We next examined the effect of FXIa on FX generation in this system. It should be noted that cleavage of the FXa substrate required the presence of both FVIIa and FX, while hydrolysis of the FXa substrate was not observed when either FVIIa or FX were omitted (S2A Fig) and that, FXIa itself did not directly activate FX or cleave the chromogenic substrate under these conditions. After the incubation of TFPIα with 2 nM FXIa for 30 min in the presence of either 25 μM Zn^2+^ or 10 μM SCP alone, or the combination of 10 μM SCP and 25 μM Zn^2+^, the addition of TFPIα decreased the generation of FXa from the baseline of 1.10±0.01 nM to 0.17±0.03, 0.40±0.06 or 0.84±0.08.2 nM, respectively (Fig 3A), suggesting that both SCP and Zn^2+^ enhanced the inactivation of TFPIα by FXIa. In the absence of FXIa, neither SCP nor Zn^2+^ affected the activity of TFPIα (S2B Fig). The incubation of TFPIα with 250 pM FXIa for 30 min in the presence of 10 μM SCP and 25 μM Zn^2+^ significantly decreased the ability of 2.5 nM TFPIα to inhibit FXa generation (Fig 3B).

We next used FXIa^ABS^ to examine whether the FXI anion-binding site (ABS), which is required for SCP-mediated enhancement of FXI activation [18], is required to mediate SCP binding to potentiate the cofactor function of SCP for the inactivation of TFPIα by FXIa. Increasing concentrations of TFPIα (0–2.5 nM) decreased the generation of FXa from the baseline of 0.8±0.01 nM to 0.07±0.008 nM in a TFPIα concentration-dependent manner (Fig 3C). In accord with our previous observations, the inclusion of 4 nM FXIa^WT^ or FXIa^ABS^ only slightly reduced the function of TFPIα. It is noteworthy that the inhibitory function of FXIa^ABS^ was equivalent to wild-type FXIa. Again, the addition of 25 μM Zn^2+^ in combination with 4 nM FXIa^ABS^ or FXIa^ABS^ significantly decreased the ability of 2.5 nM TFPIα to inhibit FXa generation. Now, in contrast to the synergistic effect of SCP in promoting the inactivation of TFPIα by FXIa^WT^, the addition of SCP to the combination of 4 nM FXIa^ABS^ and 25 μM Zn^2+^ used to pretreat TFPIα did not further increase the final rate of FXa generation, indicating that the anion-binding site of FXIa is absolutely required for the synergistic cofactor effect of SCP in the inactivation of TFPIα.

### Short-chain polyphosphates accelerate FXa-induced clotting of plasma in the presence of TFPIα and FXIa

**Fig 4:**
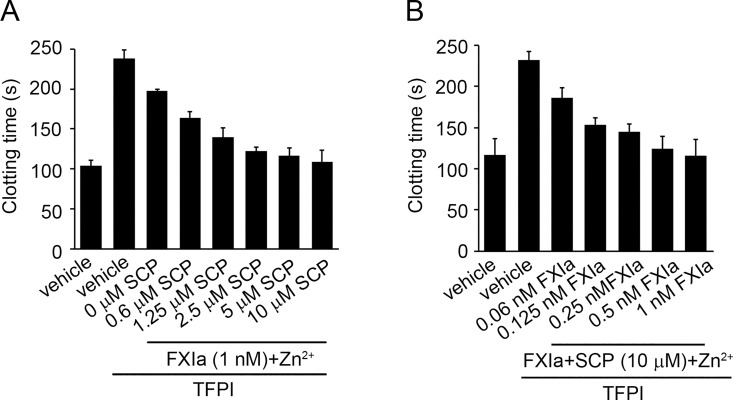
Short-chain polyphosphates accelerate the inhibition of TFPIα by FXIa. (**A**) FXa (0.5 nM)-induced clotting time of FX-depleted plasma was measured in the presence of vehicle or 5 nM TFPIα pretreated with 1 nM FXIa + 25 μM Zn^2+^ for 30 min in the presence of 0, 0.6, 1.25, 2.5,5 or 10 μM SCP. After 30 min of incubation with FXIa, aprotinin (50 μM) and polybrene (16 μM) were added to stop the reaction. (**B**) FXa (0.5 nM)-induced clotting time of FX-depleted plasma was measured in the presence of vehicle or 5 nM TFPIα pretreated with 0.06, 0.125, 0.25, 0.5 or 1 nM FXIa for 30 min in the presence of 10 μM SCP and 25 μM Zn^2+^. After 30 min of incubation with FXIa, aprotinin (50 μM) and polybrene (16 μM) were added to all samples to stop the reaction. Data are mean ± SE (n = 3).

We next determined whether FXIa degradation of rTFPIα in the presence of SCP decreased the FXa-inhibitory activity of TFPIα in plasma. Based on the fact that FXIa is able to activate FX and FV [4,6], 0.5 nM FXa was added to FX-depleted plasma in the presence of the anti-FXI antibody, 1A6, which inhibits the activation of FV and FIX by FXIa. The clotting time of recalcified FX-depleted plasma was 99.6±6.4 sec after addition of FXa (Fig 4A); this clotting time was subsequently elongated to 246±10 sec through the addition of 5 nM TFPIα. After the incubation of 5 nM TFPIα with 1 nM FXIa for 30 min, the addition of FXIa-pretreated TFPIα only increased the FXa-induced clotting time of FX-depleted plasma to 190±10 sec. Strikingly, the inhibitory function of TFPIα was abrogated following the pretreatment of TFPIα with FXIa in the presence of 10 μM SCP and Zn^2+^ (Fig 4A). Moreover, our results show that in the presence of 10 μM SCP and 25 μM Zn^2+^, even concentrations of FXIa as low as 60 pM were able to reverse the inhibitory effect of TFPIα, as evidenced by an increase in the final clotting times to 187.8±10 sec (p<0.05; Fig 4B). The addition of FXIa and SCP in the absence of TFPIα did not affect the clotting time.

### Platelet-derived short-chain polyphosphates accelerate the inhibition of platelet-derived TFPIα by FXIa

**Fig 5:**
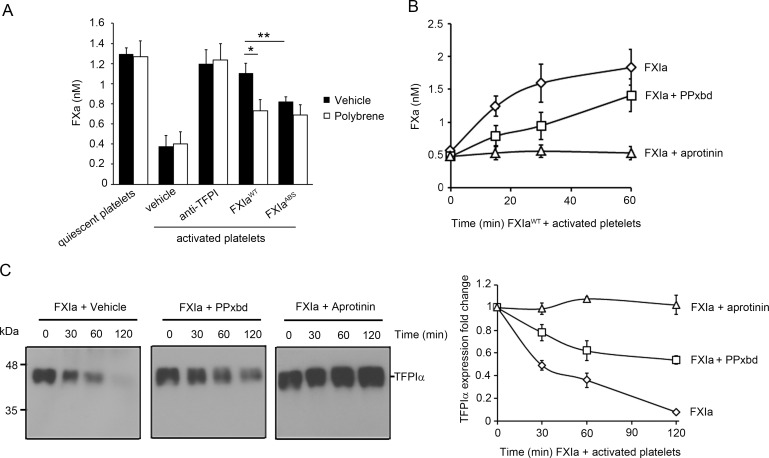
Platelet-derived short-chain polyphosphates accelerate the inhibition of platelet TFPIα by FXIa. **(A)** FXa generation following initiation with TF was determined in the absence or presence of supernatant from activated platelets. In selected experiments, the supernatant from 2×10^8^ platelets/ml was pretreated with 2 nM FXIa^WT^ or FXIa^ABS^ for 60 min in the presence or absence of polybrene (16 μM). In separate experiments, platelet supernatant from activated platelets was incubated with blocking anti-TFPI K1 and K2 antibodies (50 μM). Aprotinin (50 μM) was added to all samples to stop the reaction. Data are mean ± SE (n = 4). * *P* < 0.05 with respect to vehicle in the presence of FXIa^WT^. ** *P* < 0.05 with respect to vehicle in the presence of FXIa^WT^. Mann-Whitney *U* test was used for statistical comparisons. **(B)** FXa generation following initiation with TF was determined in the presence of supernatant from activated platelets pretreated with 2 nM FXIa in the presence or absence of PPxbd (250 μg/ml) for 0, 15, 30 or 60 min. Aprotinin (50 μM) was added to all samples to stop the reaction. Data are mean ± SE (n = 3). **(C)** Supernatant from activated platelets (2.5×10^8^) was incubated with 5 nM FXIa for 0, 30, 60 or 120 min in the absence or presence of PPxbd (250 μg/ml) or aprotinin. The extent of TFPI present in the supernatant was analyzed by western blotting with a polyclonal anti-TFPI antibody. The bands were quantified by densitometry. Data are mean ± SE (n = 3)

We next compared the effect of supernatant from resting versus activated platelets on FXa generation. Our results show that the addition of TF-FVIIa (50 pM) to FX (100 nM) resulted in a baseline FXa generation of 1.23±0.06 nM in the presence of supernatant from resting platelets. The addition of supernatant from activated platelets reduced the generation of FXa to 0.37±0.011 nM (Fig 5A). This inhibitory effect of the supernatant from activated platelets was reversed back to the baseline levels by the addition of blocking anti-TFPIα Abs, suggesting that TFPIα secreted from activated platelets was responsible for inhibition of FXa generation in this system. A similar effect was observed when the supernatant was pretreated with 2 nM FXIa^WT^ for 1 hr, resulting in an increase in the final FXa generation rate to 1.11±0.10 nM. This effect was lost when 2 nM FXIa^ABS^ was used, as FXIa^ABS^ has a reduced capacity to bind polyphosphates, resulting in a depressed FXa final generation rate of 0.82±0.05 nM. Moreover, our data show that use of polybrene, which binds to polyanions and therefore should reduce the cofactor activity of SCP, reversed the inhibitory effect of FXIa^WT^ on the supernatant of activated platelets, resulting in a decrease in the final generation rate from 1.11±0.10 pmoles/min to 0.68 ± 0.01 nM (Fig 5A).

Time course experiments in the presence of a polyphosphate-binding protein (PPxbd) were next performed in order to test the specificity of SCP in promoting the inactivation of TFPIα by FXIa. The incubation of supernatant from activated platelets with 2 nM FXIa increased the generation of FXa from the baseline of 0.56 ± 0.03 nM to 0.18 ± 0.028 nM in a time-dependent manner (Fig 5B). The incubation of supernatant from activated platelets with FXIa in the presence of PPxbd increased the generation of FXa from the baseline of 0.48 ± 0.03 nM to 0.14± 0.025 nM (Fig 5B), suggesting that SCP present in the activated platelet supernatant acts as an endogenous cofactor to drive the inactivation of TFPIα by FXIa and promote FXa and eventually thrombin generation. The same result was obtained in the presence of supernatant from platelets activated with either thrombin or thrombin and the platelet GPVI-specific agonist CRP (S3 Fig). Western blot analyses of TFPIα in the platelet supernatant demonstrated that the presence of PPxbd decreased the rate of TFPIα proteolysis by FXIa (Fig 5C). The presence of aprotinin completely blocked the capacity of FXIa to either inhibit TFPIα activity (Fig 5B) or TFPIα proteolysis (Fig 5C).

### Short-chain polyphosphates accelerate the inhibition of TFPIα by FXIa in plasma

**Fig 6:**
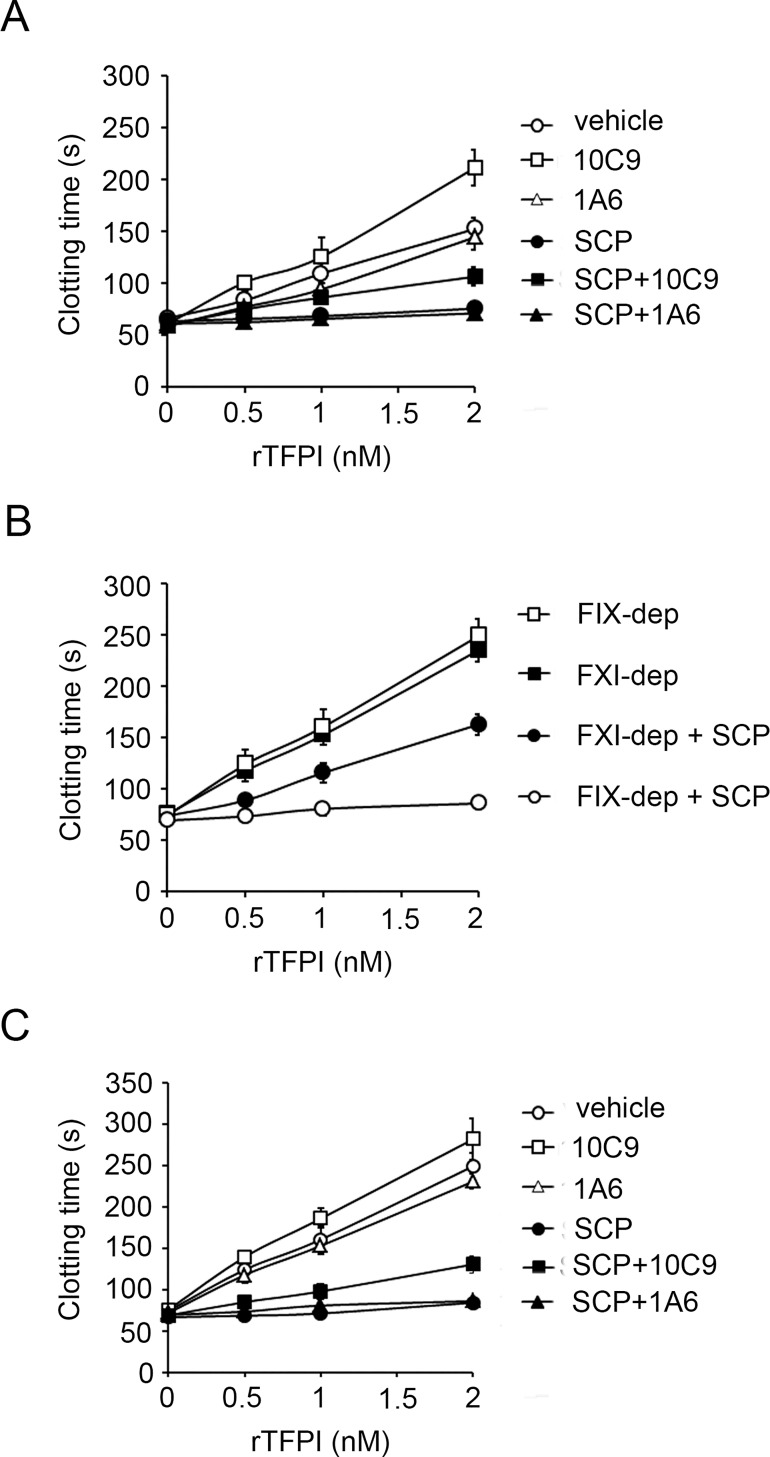
FXI is required for short-chain polyphosphates to inhibit the anticoagulant effect of TFPIα in plasma. (**A**) TF-induced clotting times were measured in normal plasma in the presence of increasing concentrations of TFPIα in the absence (○, ◻, △) or presence of 5 μM SCP (●, ■, ▲). Plasma was pretreated with vehicle (○,●), 20 μg/ml 1A6 (△, ▲), or 50 μg/ml 10C9 (◻,■). (**B**) TF-induced clotting times were measured in FIX-depleted plasma (FIX-dep) (■,●) or FXI-depleted plasma (FXI-dep) (◻,○) in the presence of increasing concentrations of TFPIα in the absence (■,◻) or presence of 5 μM SCP (○,●). (**C**) TF-induced clotting times were measured in FIX-depleted plasma in the presence of increasing concentrations of TFPIα in the absence (○, ◻, △) or presence of 5 μM SCP (●, ■, ▲). Plasma was pretreated with vehicle (○,●), 20 μg/ml 1A6 (△, ▲), or 50 μg/ml 10C9 (◻,■). Data are mean ± SE (n = 3).

We measured the effect of SCP on the inhibition of TFPIα anticoagulant activity by FXIa. In the presence of 8 pM TF, the addition of 2 nM TFPIα increased the clotting time of recalcified plasma from 66.6±3.7 sec to 152.8±11.2 sec. This anticoagulant effect of TFPIα was reduced by the addition of FXIa and 5μM SCP to the plasma, resulting only in a small increase in clotting time to 75.7±6.7 sec (Fig 6A), in accord with previously studies [16]. TFPIα prolonged the clotting time of plasma preincubated with a FXIa active site domain-neutralizing antibody, 10C9, to 106.7±8.6 sec in the presence of SCP (Fig 6A). In contrast, TFPIα did not significantly prolong the clotting time of plasma preincubated with an anti-FXI antibody, 1A6, which inhibits the activation of FV by FXIa and the activation of FIX by FXIa in the presence of SCP (Fig 6A).

In the presence of TF, addition of 2 nM TFPIα also increased the clotting time of FXI-depleted plasma or FIX-depleted plasma from 75±6 sec to 236±8 sec and 74.3±4.1 sec to 250±16.6 sec, respectively (Fig 6B). TFPIα prolonged the clotting time of FXI-depleted plasma from 74±6 sec to 163±10 sec in the presence of SCP (Fig 6B). In contrast, TFPIα did not significantly prolong the clotting time of FIX-depleted plasma in the presence of SCP, resulting only in a small increase in clotting time to 86.06±4.6 (Fig 6B). TFPIα prolonged the clotting time of FIX-depleted plasma preincubated with 10C9 to 135.6±10.3 sec in the presence of SCP (Fig 6C). In contrast, TFPIα did not significantly prolong the clotting time of FIX-depleted plasma preincubated with 1A6 in the presence of SCP (Fig 6C).

## Discussion

FXI appears to be the only coagulation factor of the contact pathway required for hemostasis. While the primary substrate for FXIa is FIX, FXIa also promotes thrombin generation by direct activation of FX, FV and FVIII [4–6]. Moreover, we recently demonstrated that FXIa binds to TFPIα and inhibits the anticoagulant activity of TFPIα [14]. Polyphosphate released from platelet dense granules has been shown to bind to FXI and enhance FXI autoactivation, FXI activation by thrombin, and FV activation by FXIa [7,9]. These observations suggest that the hemostatic activity of FXIa could be enhanced by activated platelets. Here we show that synthetic as well as platelet-derived short-chain polyphosphates serve as a potent cofactor to enhance the inactivation of TFPIα by FXIa.

Polyphosphate of the size released from platelets (SCP) and even shorter polymers have been shown to inhibit the effect of TFPIα on TF- or FXa-induced plasma clotting [15,16]. We observed that SCP enhanced the ability of FXIa to block the anticoagulant effect of TFPI in plasma in a FIX-independent manner. It is known that when FVa is bound to FXa in the presence of prothrombin, TFPIα is not able to inhibit FXa [23]. Our data would suggest that the release of SCP from platelets acts to block the activity of TFPIα in plasma by enhancing the activation of FV by thrombin, FXa and FXIa in concert with the capacity of SCP to enhance the direct inhibition of TFPIα by FXIa. TFPIα is known to bind to polyanions such as the cell-surface glycosaminoglycan, heparan sulfate, through its basic C-terminal region [24]. Our result shows that SCP is able to bind to TFPIα. The fact that heparin blocks the binding of SCP to TFPIα suggests that TFPIα binds to SCP through its basic C-terminal region. We previously demonstrated that the inactivation of HUVEC-derived TFPIβ by FXIa resulted in the loss of the K1 domain of TFPIβ and promotion of TF-dependent fibrin formation [14]. Interestingly, we found that addition of SCP did not enhance the capacity of FXIa to block the anticoagulant activity of HUVEC-derived TFPIβ (S4 Fig). TFPIβ is comprised of the K1 and K2 domains attached to a glycosylphosphatidylinositol-anchored C-terminal moiety and does not have a basic C-terminal region. Therefore, it appears likely that TFPIβ cannot bind to platelet-derived short-chain polyphosphate, explaining why SCP does not serve as a cofactor for the inhibition of TFPIβ by FXIa. We have previously shown that the inhibition of TFPIα by FXIa was enhanced in the presence of Zn^2+^ [14]. Here we show that Zn^2+^ also enhances the capacity of SCP to increase the inhibition of TFPIα by FXIa. Polyphosphates are known to form chelate complexes with metal ions [25]. This is noteworthy in light of the fact that the formation of ellagic acid-metal ion complexes has been shown to increase the procoagulant activity of ellagic acid [26]. It has been reported that immobilized transition metal ions stimulate activation of the contact pathway [27]. Previous studies have suggested that FXI binds to platelets in a Zn^2+^-dependent manner [28] and that Zn^2+^ enhances FXI autoactivation and FXI activation by thrombin in the presence of glycosaminoglycans [29]. Taken together, these data suggest that the presence of Zn^2+^ may play an important role in the regulation of FXIa activity by SCP.

Clinical studies have shown a direct relationship between high levels of FXI and thrombosis in humans. Interestingly, the inhibition of FXI activation has been shown to be beneficial in a mouse model of TF-induced pulmonary embolism [30]. Furthermore, we have shown that in a primate model of thrombosis, the inhibition of FXI reduced intraluminal thrombus growth initiated by TF [31]. Moreover, reducing FXI levels in patients undergoing unilateral total knee arthroplasty has been shown to be an effective method for the prevention of postoperative venous thromboembolism [32]. Interestingly, it has been shown that platelet-derived polyphosphate potentiates the activation of FXI independent of FXIIa in human blood under flow conditions, which requires the participation of the extrinsic pathway [19]. Also, for human FXI, Lys529, Arg530, and Arg532 in the catalytic domain form one of the ABS and is required for polyphosphate-mediated enhancement of FXI activation [18]. Our results here demonstrate that this anion-binding region is required for SCP to enhance the capacity of FXIa to inhibit TFPIα. This mechanism may partially explain why the loss of the FXI catalytic domain ABS promotes an antithrombotic effect in a mouse model of thrombosis. These results also raise the possibility that the role of FXI in promoting thrombus growth in the presence of TF is not restricted to activation of FIX, but may in part involve the capacity of FXIa to inhibit TFPIα in the presence of platelet-derived polyphosphate.

TFPI seems to have a pathologic role during hemostasis in patients with hemophilia. TFPI-blocking antibodies shorten the clotting time of FVIII-deficient plasma, and administration of TFPI-blocking antibodies improves hemostasis in hemophilic mice [33]. Also, the inherited east Texas bleeding disorder, which leads to the formation of the TFPIα-FV complex, is characterized by a 10-fold increase in plasma TFPIα, resulting in a bleeding disorder [34]. It has been difficult to predict bleeding tendencies in FXI-deficient patients, as symptoms are poorly correlated with plasma FXI activity, and are highly variable even among patients with similar FXI levels. It has been reported that thrombin generation measured in platelet-rich plasma in the presence of TF and contact pathway inhibitors is able to identify which FXI-deficient patients present with a bleeding phenotype [35], suggesting that platelets may play an important role in the regulation of FXI in hemostasis. Interestingly, in another study, patients with severe FXI deficiency with a history of bleeding had higher levels of TFPI than asymptomatic patients [36]. Prediction of bleeding in asymptomatic FXI-deficient patients poses an important dilemma to surgeons, and may also become a clinical issue in the future with the dawn of antithrombotic FXIa inhibitors in development that block en masse the enzymatic action of FXIa [37]. Our results demonstrate that the release of SCP from activated platelets acts as a cofactor for the inhibition of TFPIα, suggesting that activated platelets in a hemostatic plug may contribute to the hemostatic role of FXI not only by enhancing the activation of FXI by thrombin and the activation of FV and FX by FXIa, but also by promoting thrombin generation through neutralization of TFPI.

## Supporting Information

S1 Fig Characterization of the interaction between heparine and TFPIα. 96-well plates were coated with 5 μg/ml TFPIα (○,●) or BSA (◻) and increasing concentrations of biotinylated-polyphosphate (bio-polyP) (◻, ○,●) was added to selected wells. Selected experiments were done in the presence of 10 U/ml (●). Binding was detected with streptavidin-HRP. Data are mean ± SE (n = 3).
(TIF)

S2 Fig FXa generation in purified systems. **(A)** FXa generation by the TF-FVIIa complex in the presence of absence of FXIa (2 nM) preincubated with aprotinin (50 μM) was measured. **(B)** FXa generation by the TF-FVIIa complex in the presence of different concentrations of TFPIα in the absence or presence of 25 μM Zn^2+^and 10 μM platelet-size polyphosphate (SCP) was measured.
(TIF)

S3 Fig Platelet activated by thrombin or thrombin and CRP derived SCP and accelerated the inhibition of platelet TFPIα by FXIa. FXa generation following initiation with TF was determined in the absence or presence of supernatant from platelets activated with either thrombin (Thr) or Thr and CRP. In selected experiments, the supernatant from 2×10^8^ platelets/ml was pretreated with 2 nM FXIa for 30 min in the presence or absence of PPXbd.
(TIF)

S4 Fig Inhibition of TFPI by FXIa on the surface of endothelial cells in the presence of SCP. HUVECs were grown to confluence in 96-well plates and incubated for 3 hrs in serum-free medium with 0.3% BSA with TNFα (0.5 nM). Subsequently, 15 nM FXIa in the presence or absence of SCP (10 μM). or an anti-TFPI antibody (50 μg/ml) was added for 1 hr followed by incubation with 50 μM aprotinin for 10 min at 37oC, washed with HEPES-buffered saline and incubated for 30 mins at 37oC with 0.1nM FVIIa, 100 nM FX in HBS-Ca2+ and 0.3% BSA. HEPES-buffered saline containing 100 mM EDTA was added to stop the reaction. The chromogenic FXa substrate, Spectrozyme Xa, was added in order to determine the initial rate of substrate hydrolysis. Rates of Spectrozyme FXa hydrolysis measured at 405nm were converted to FXa concentrations using a standard curve.
(TIF)
