# Supplementary figures and images for: Platelet-Derived Short-Chain Polyphosphates Enhance the Inactivation of Tissue Factor Pathway Inhibitor by Activated Coagulation Factor XI
Source: PLoS One. 2016 Oct 20;11(10):e0165172. doi: 10.1371/journal.pone.0165172 (PMC5072614; doi:10.1371/journal.pone.0165172)

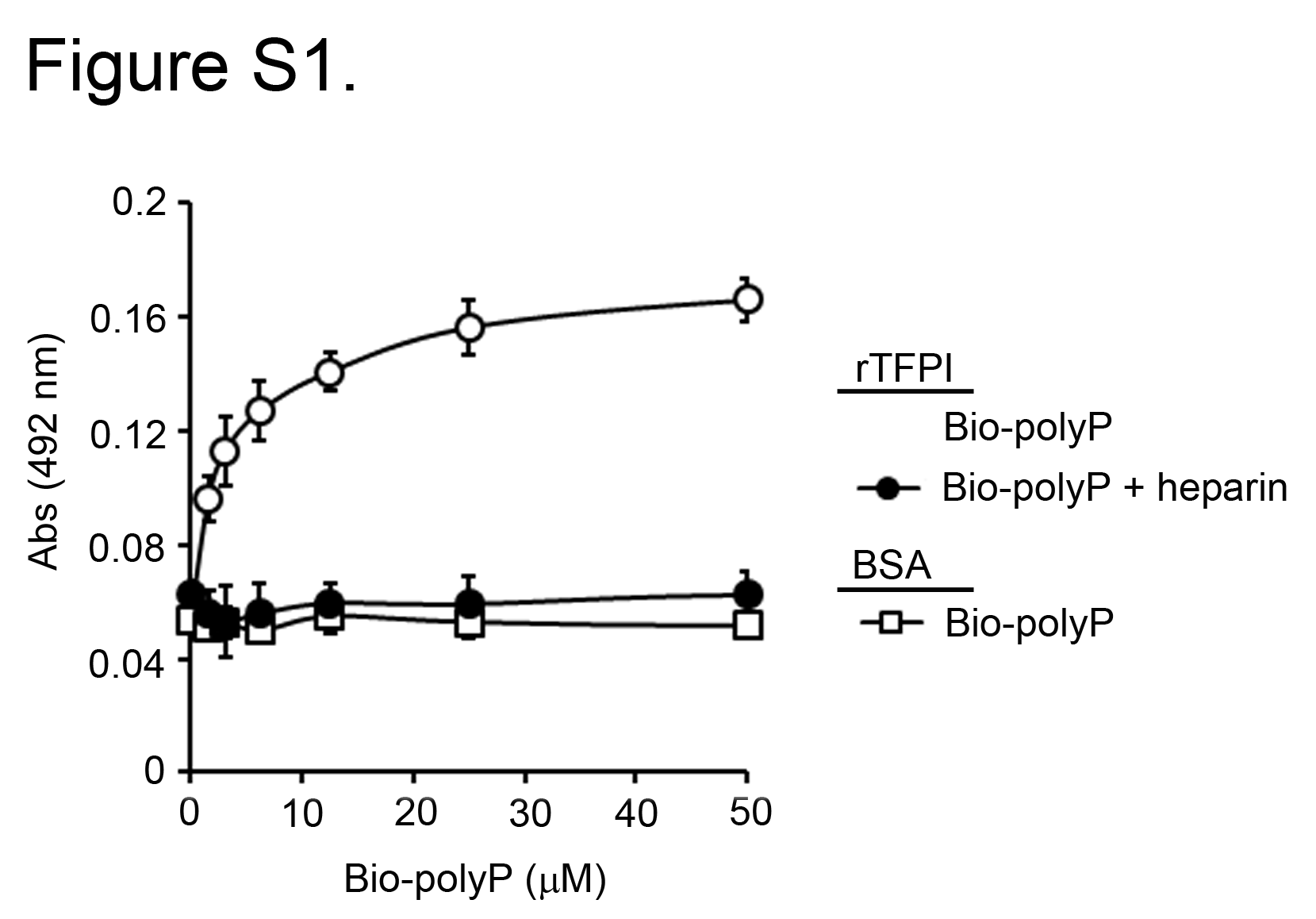

Supplement: S1 Fig — 96-well plates were coated with 5 μg/ml TFPIα (○,●) or BSA (◻) and increasing concentrations of biotinylated-polyphosphate (bio-polyP) (◻, ○,●) was added to selected wells. Selected experiments were done in the presence of 10 U/ml (●). Binding was detected with streptavidin-HRP. Data are mean ± SE (n = 3). (TIF) [file pone.0165172.s001.tif]

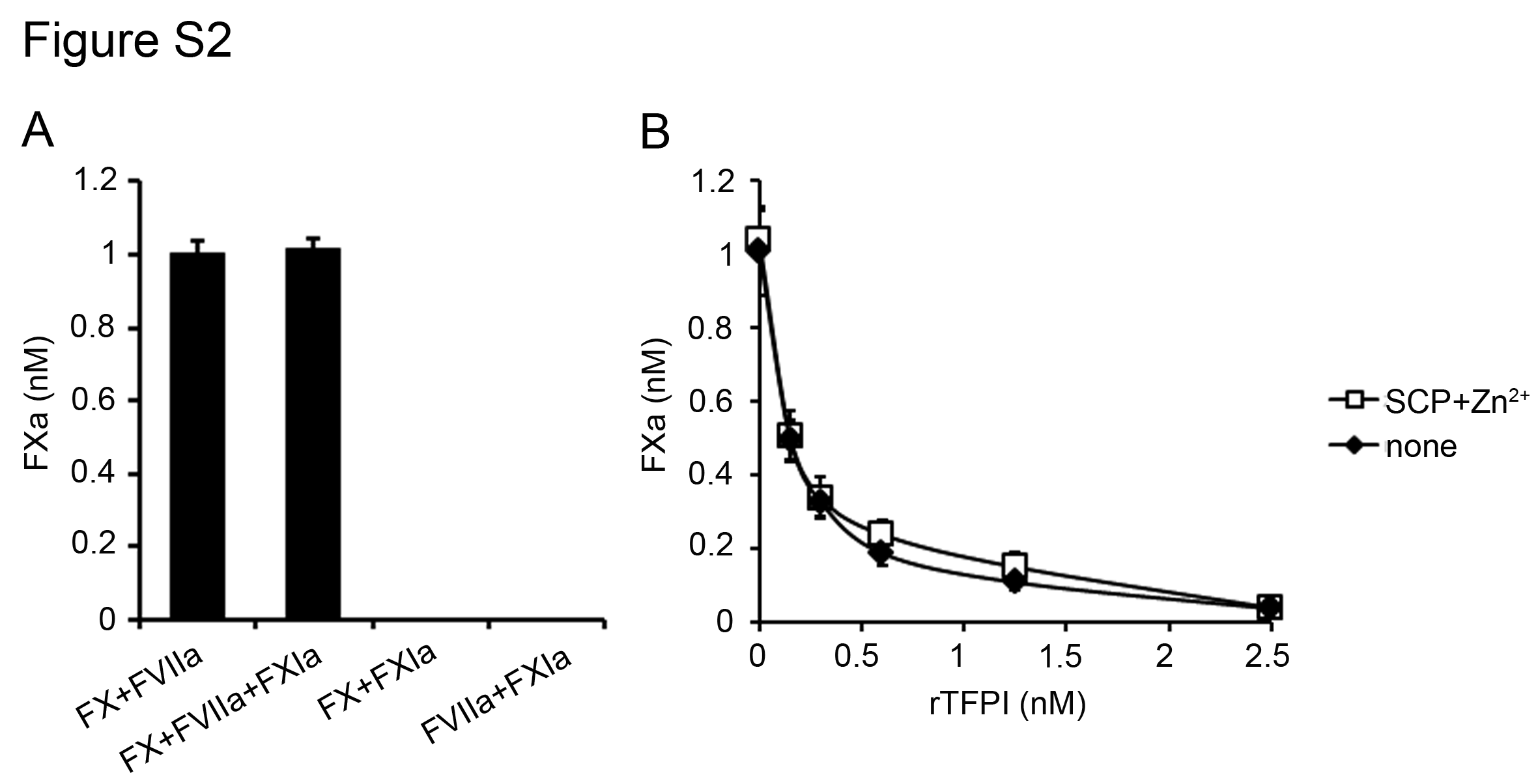

Supplement: S2 Fig — (A) FXa generation by the TF-FVIIa complex in the presence of absence of FXIa (2 nM) preincubated with aprotinin (50 μM) was measured. (B) FXa generation by the TF-FVIIa complex in the presence of different concentrations of TFPIα in the absence or presence of 25 μM Zn2+and 10 μM platelet-size polyphosphate (SCP) was measured. (TIF) [file pone.0165172.s002.tif]

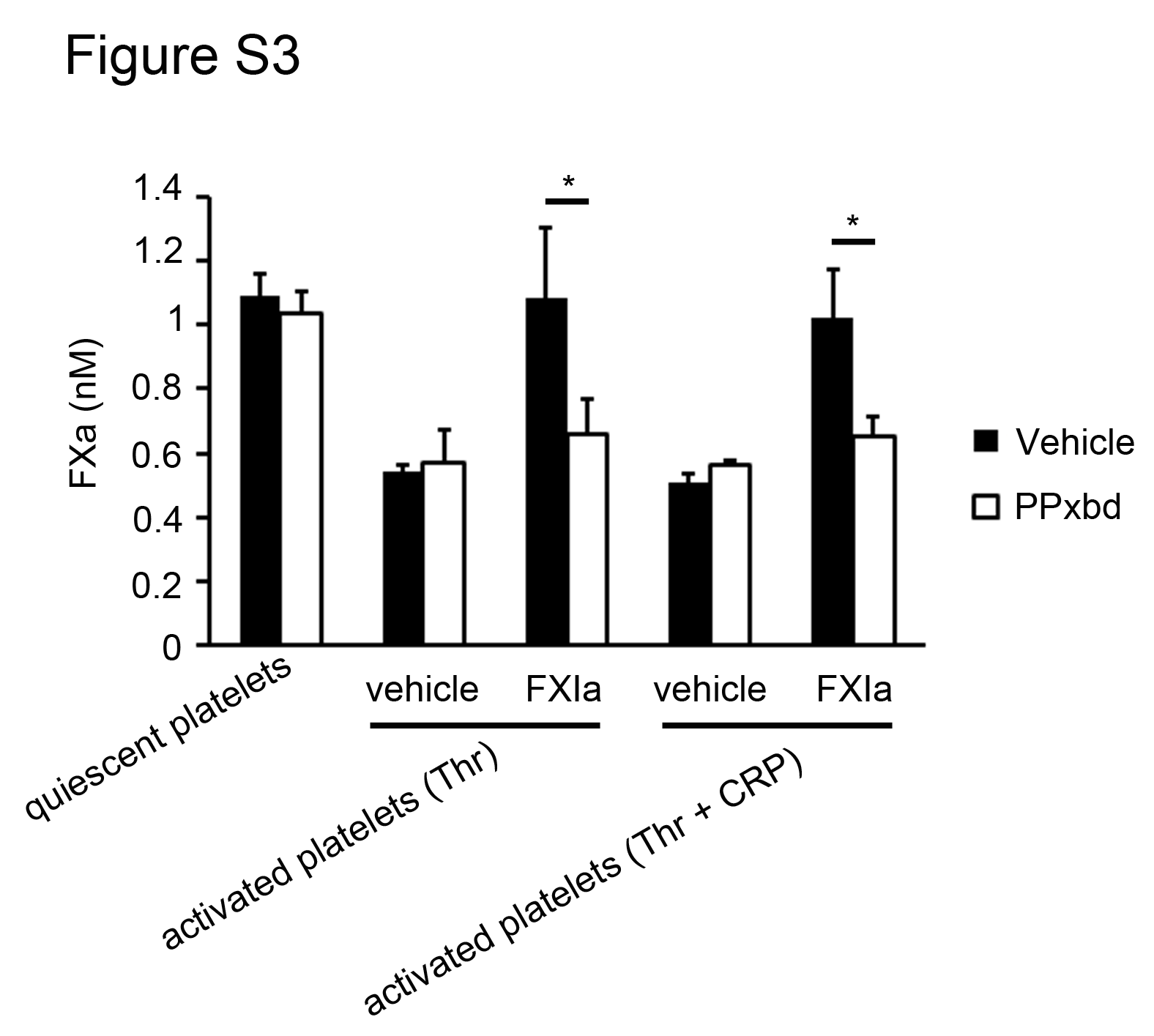

Supplement: S3 Fig — FXa generation following initiation with TF was determined in the absence or presence of supernatant from platelets activated with either thrombin (Thr) or Thr and CRP. In selected experiments, the supernatant from 2×10^8^ platelets/ml was pretreated with 2 nM FXIa for 30 min in the presence or absence of PPXbd. (TIF) [file pone.0165172.s003.tif]

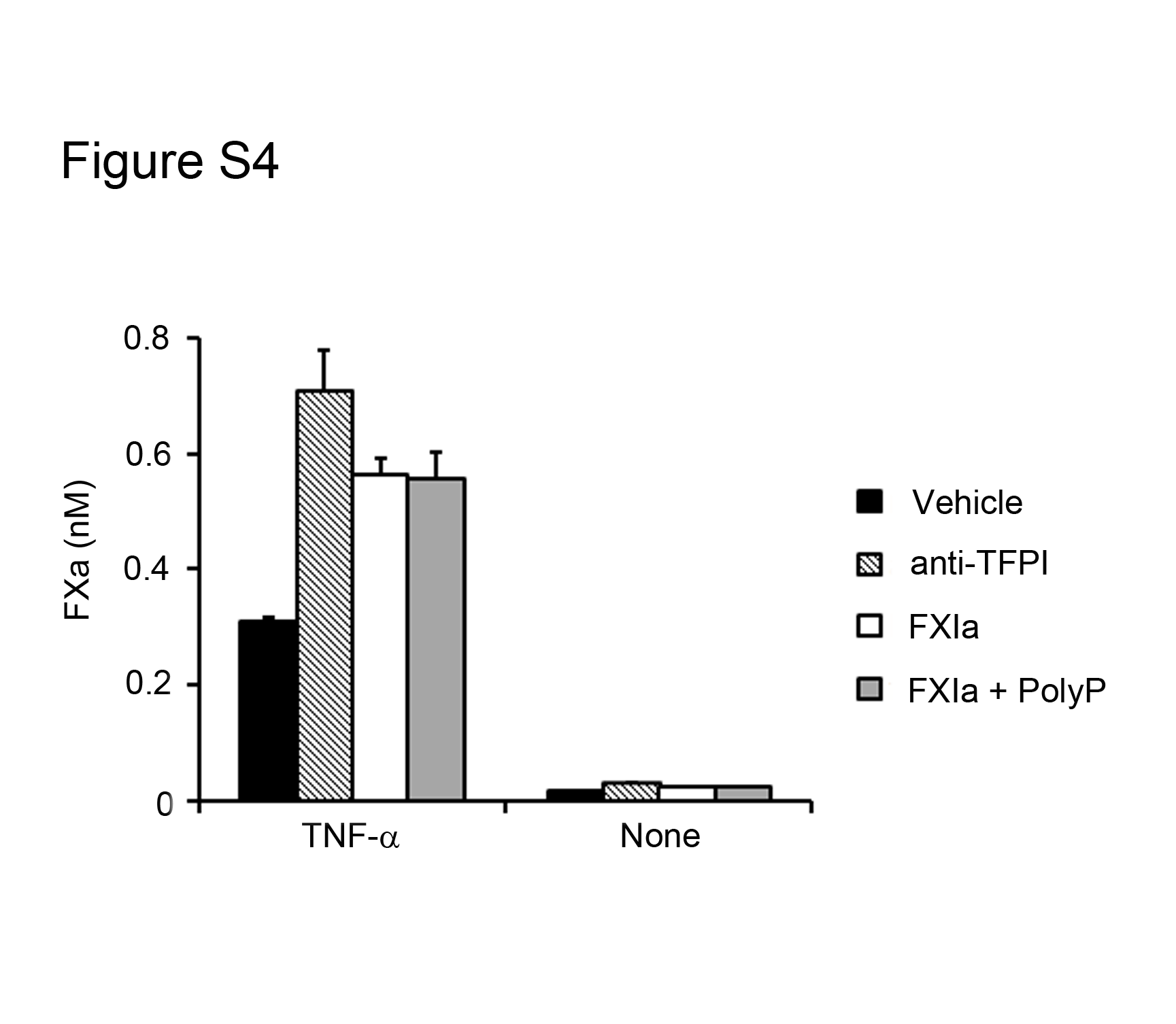

Supplement: S4 Fig — HUVECs were grown to confluence in 96-well plates and incubated for 3 hrs in serum-free medium with 0.3% BSA with TNFα (0.5 nM). Subsequently, 15 nM FXIa in the presence or absence of SCP (10 μM). or an anti-TFPI antibody (50 μg/ml) was added for 1 hr followed by incubation with 50 μM aprotinin for 10 min at 37oC, washed with HEPES-buffered saline and incubated for 30 mins at 37oC with 0.1nM FVIIa, 100 nM FX in HBS-Ca2+ and 0.3% BSA. HEPES-buffered saline containing 100 mM EDTA was added to stop the reaction. The chromogenic FXa substrate, Spectrozyme Xa, was added in order to determine the initial rate of substrate hydrolysis. Rates of Spectrozyme FXa hydrolysis measured at 405nm were converted to FXa concentrations using a standard curve. (TIF) [file pone.0165172.s004.tif]
